# Using Ontario's "Telehealth" health telephone helpline as an early-warning system: a study protocol

**DOI:** 10.1186/1472-6963-6-10

**Published:** 2006-02-15

**Authors:** Elizabeth Rolland, Kieran M Moore, Victoria A Robinson, Don McGuinness

**Affiliations:** 1 Ontario Syndromic Surveillance Project, Kingston, Canada; 2 Infectious Disease Epidemiology Unit, London School of Hygiene and Tropical Medicine, London, UK; 3 Department of Emergency Medicine and Community Health and Epidemiology, Queen's University, Canada; 4 Institute of Medical Science, University of Toronto, Toronto, Canada

## Abstract

**Background:**

The science of syndromic surveillance is still very much in its infancy. While a number of syndromic surveillance systems are being evaluated in the US, very few have had success thus far in predicting an infectious disease event. Furthermore, to date, the majority of syndromic surveillance systems have been based primarily in emergency department settings, with varying levels of enhancement from other data sources. While research has been done on the value of telephone helplines on health care use and patient satisfaction, very few projects have looked at using a telephone helpline as a source of data for syndromic surveillance, and none have been attempted in Canada. The notable exception to this statement has been in the UK where research using the national NHS Direct system as a syndromic surveillance tool has been conducted.

**Methods/design:**

The purpose of our proposed study is to evaluate the effectiveness of Ontario's telephone nursing helpline system as a real-time syndromic surveillance system, and how its implementation, if successful, would have an impact on outbreak event detection in Ontario. Using data collected retrospectively, all "reasons for call" and assigned algorithms will be linked to a syndrome category. Using different analytic methods, normal thresholds for the different syndromes will be ascertained. This will allow for the evaluation of the system's sensitivity, specificity and positive predictive value. The next step will include the prospective monitoring of syndromic activity, both temporally and spatially.

**Discussion:**

As this is a study protocol, there are currently no results to report. However, this study has been granted ethical approval, and is now being implemented. It is our hope that this syndromic surveillance system will display high sensitivity and specificity in detecting true outbreaks within Ontario, before they are detected by conventional surveillance systems. Future results will be published in peer-reviewed journals so as to contribute to the growing body of evidence on syndromic surveillance, while also providing an non US-centric perspective.

## 

According to the Oxford Handbook of Public Health Practice[1], two of the principal objectives of an effective surveillance system are to "give *early warning *changes of incidence," and "detect *outbreaks *early." Unfortunately, the reality of public health practice is that monitoring agencies such as public health units routinely fall short of these objectives, whether it is due to lags/cuts in the passive surveillance communication between physicians and public health agencies [2-4], or because of delays in laboratory confirmation[5,6]. This is even more of an issue in large and remote areas such as Northern Ontario.

A new era of surveillance research is attempting to address these issues while taking advantage of available data. Electronic data captured at the point of care provides an efficient means for the conversion of clinical data to surveillance information. This has resulted in a new area of research called syndromic surveillance.

Syndromic surveillance is a newly emerging field in the science of epidemiological surveillance. Its growth has been encouraged in large part in the United States as a response to potential bioterrorism threats. In effect, it is a complementary surveillance system, in that it can provide prediagnostic data to rapidly detect infectious disease outbreaks before they are detected through conventional surveillance methods.

The Walker Report[7] highlighted Canadian post-SARS interest in syndromic surveillance as a method of increasing Ontario's capacity to manage communicable diseases adequately. An important feature of syndromic surveillance systems is that they rely on existing data streams. In other words, it does not require the development of new datasets (and the challenges surrounding this), but rather makes use of available data and increases communications within the public health system. Data streams that are being investigated as part of the larger syndromic surveillance picture include:

- Over The Counter (OTC) drug sales [8-12],

- emergency department visits (coded either by ICD code or by chief complaint) [13,14],

- emergency (911) calls[15],

- ambulance dispatch[10],

- patient transfers[16],

- school/work absenteeism records[10],

- telephone medical helpline calls (e.g. *NHS Direct in the UK*, Ontario *Telehealth*) [10,17-22],

- insurance/HMO claim data[23].

The science of syndromic surveillance is still very much in its infancy. While a number of syndromic surveillance systems are being evaluated in the US, very few have had success thus far in predicting an infectious disease event.

However, syndromic surveillance can and has been able to assist in "determin[ing] the size, spread and tempo of an outbreak after it has been detected," [24]. It can also theoretically be useful in times of calm, "providing reassurance that a large-scale outbreak is not occurring," [23].

Currently, most systems are primarily based in an emergency department/room setting, where they rely on either chief complaint information recorded at onset of contact with a syndromic surveillance source (e.g. triage nurse), or ICD9/10 codes, following diagnosis by the physician. Although studies have shown that using ICD codes results in better sensitivity, positive predictive value and specificity[25,26], there is often a lag between the contact with the case, and the coding of his/her information into an ICD case. In some settings, this can take up to a week[23,27]. Consequently, the majority of emergency department syndromic surveillance systems rely primarily on chief complaint for the classification of visits into syndromes (symptom categories).

To date, the majority of syndromic surveillance systems have been based primarily in emergency department settings, with varying levels of enhancement from other data sources, such as the ones listed earlier. While research has been done on the value of telephone helplines on health care use and patient satisfaction[28], very few projects have looked at using a telephone helpline as a source of data for syndromic surveillance, and none have been attempted in Canada. The notable exception to this statement has been in the UK where research using the national NHS Direct system as a syndromic surveillance tool has been conducted.

The NHS Direct national telephone helpline was established in the UK in 1998 to provide both health information as well as medical referral to callers. The country has 21 regional calling sites in England and Wales[29]. In order to address calls pertaining to medical referrals, a clinical decision support software package (NHS CAS) with over 200 clinical algorithms is used. Each of these 200+ algorithms has a set of questions attached to them; their purpose is to ascertain the caller's symptoms and provide them with the most appropriate advice and course of action[28].

Since 1999, researchers in the UK have been retrospectively and prospectively investigating the use of NHS Direct as a syndromic surveillance method for a number of syndromes[18,19,28,30], including influenza[21,30,31] and gastrointestinal illness[22]. To date, their results have been optimistic about the long-term use of NHS Direct as an "early warning system," but acknowledge that work continues to be required to fine-tune the system.

Due to its central database structure, Ontario's Telehealth system should theoretically be better suited as a source for a syndromic surveillance system than its UK counterpart. While the UK system relies on regional calling centers with local databases to deal with call volumes from their proprietary calling region, the Ontario Telehealth System has four call centres that manage the call loads for the province as a whole, but decision rules are identical for all four centers, and all data are stored within one central database. This ensures that triage algorithms are the same for all regions, which is not the case in the UK.

## Background

The Ontario Teleheath System (henceforth referred to as "Telehealth") is a tollfree telephone helpline provided by the Ontario Ministry of Health and Long-term Care, and is available to all residents of Ontario. The Telehealth program was initiated as a pilot in 2001 in the Greater Toronto (416 and 905 calling areas) and Northern areas (705 calling area). The program became province-wide at the end of 2001. The Telehealth services are provided by a private contractor (currently "Clinidata") hired by the Ministry of Health and Long-Term Care.

Telehealth operates 24 hours a day, 7 days a week and all calls are answered by registered nurses who have at least three years of recent clinical experience[32]. Calls are answered in both official languages, but translators are available via three-way call within 60 seconds for 110 languages[32]. The most commonly requested languages other than English and French are Mandarin, Cantonese, Farsi, Italian and Portuguese[32,33]. An average of 3,100 calls are recorded daily[33], with the highest call volumes occurring during the evenings and holidays. Seasonally, the busiest months are the winter months (January-March), which coincide with cold and influenza seasons[34]. The average call length is approximately 10 minutes[32], and calls are classified in one of five categories[35]:

1) priority (call 911 immediately);

2) emergent (see physician within hours – routinely directed to a hospital emergency department);

3) urgent (contact family physician within 24 hours);

4) referral 72 (contact family physician with 72 hours);

5) self-care.

According to information on the Ontario Ministry of Health and Long-Term Care website, the volume of calls attributed to these different categories is as follows: "about 43 per cent of callers received self-care advice; 35 per cent if callers were advised to visit their physician; 14 per cent were referred to a hospital emergency department; two per cent were considered urgent calls and were connected to 911," [33].

To date, 40% of callers have been parents, primarily mothers, calling about symptoms being displayed by their children. Overall, the top five reported symptoms have been: nausea/vomiting, abdominal pain, fever in children aged 3 months-3 years, cough/cold, and rash[32].

Currently, Ontario has a broad syndromic surveillance agenda, which includes pilot studies of over-the-counter drug sale monitoring[10], and emergency department visits[17]. Telehealth is expected to be a part of this overarching surveillance strategy, supplementing current syndromic surveillance systems, as well as, potentially identifying call volume and type aberrations before they also occur in hospital emergency departments.

## Research Methods/design

Using data collected by Clinidata as part of the Ontario Telehealth program since December 2001, the effectiveness of Telehealth as a real-time surveillance system will be investigated. As no research has been done in this area in Canada, and international research to date is very limited, the development of this translational tool is innovative and will add significantly to the limited state of knowledge on syndromic surveillance.

This project will have both a retrospective and a prospective component. In the first stage, our research will be retrospective in nature. Using data collected retrospectively by Clinidata, we will link all "reasons for call" ("chief complaint") and assigned algorithms to one of the following syndromes: gastrointestinal, constitutional, respiratory, rash, hemorrhagic, botulinic, neurological, and other (Table 1). These syndromes are identical to those currently used by the Ontario Syndromic Surveillance Pilot Project (also known as QUESST). This will be done in conjunction with QUESST, using their recent expertise in the mapping of health algorithms and chief complaint to these syndromes.

Following this step, analyses will be carried out to determine normal thresholds for the different syndromes. There are many different methods documented in the literature, including, but not limited to, cumulative sums[36,37], control charts[38], recursive least squares[39,40], and upper confidence limits, the latter which are the preferred method for NHS Direct research[18]. Using knowledge of past outbreak activity such as SARS and Norwalk-type outbreaks, the validity and reliability of the data, as well as, the sensitivity, specificity and positive predictive values of these different methods will be evaluated in order to ascertain which statistical method best applies to these data.

Once thresholds have been established and evaluated, the prospective monitoring of syndromic activity will take place. This will be done both temporally and spatially. The decision to pursue both types of analyses is due to Ontario's large geographic size, combined with the uneven distribution of population. Temporal trends, usually measured through one-dimensional scan statistics[36,41,42] are of interest to monitor general patterns of symptoms. This is of particular interest for unusual events where a few cases would result in a likely alert (e.g. anthrax). Spatial clustering analysis, using spatial scan statistics, have been adapted for infectious disease surveillance[36,43,44]. This statistical method will be used to ascertain unusual activity in specific geographic areas. For example, this will allow for the monitoring of underserviced areas such as northern Ontario where infectious disease surveillance is limited and where, coincidentally, use of Telehealth is proportionally high. This statistical method relies on a geographic region's expected counts rather than on its population distribution.

It is critical that new and emerging surveillance systems implement process evaluation targets within its logic model to monitor its effectiveness and progress.

In order to meet this need, we plan on implementing the Centers for Disease Control and Prevention "Framework for Evaluating Public Health Surveillance Systems for Early Detection of Outbreaks," [45]. As part of the routine evaluation of this surveillance system, it is our intention to compare syndromic surveillance activity monitored by the Telehealth Project (both retrospectively and prospectively) with emergency department syndromic surveillance activity. At present, the Ontario Emergency Department Syndromic Surveillance System only monitors hospitals located in Kingston, Ontario.

Consequently, this portion of our evaluation is only planned for Telehealth calls made in the Kingston, Frontenac, Lennox and Addington catchment area. As other hospitals are added to the ED Syndromic Surveillance Project, they will be included in the comparative evaluation of Ontario Telehealth and Emergency Department Syndromic Surveillance.

The last step of the evaluation of Telehealth as an early warning system will be an economic analysis that will attempt to ascertain whether Telehealth provides medical and non-medical cost savings, both in terms of monitoring, and public health surge capacity planning.

## Discussion

The use of Telehealth as an early-warning syndromic surveillance system will promote communication between the acute care sector and the public health sector of the health care system, by relying on the acute care system as a frontline source of information for the more effective planning and management of public health resources.

This system has the potential of identifying outbreaks (such as pandemic influenza and gastrointestinal illness) and other adverse health events (such as bioterrorist attacks) before the acute care and public health sectors would be made aware of them through established avenues. Furthermore, this would improve the commonly accepted lag between identification of an event and reporting of the event to the proper authorities.

This system, because of its automated and real-time nature would immediately identify aberrations in call types and would alert the proper authorities (including acute care facilities and public health) immediately.

Secondly, with its province-wide focus, it will potentially remedy some of the systemic failures identified by Justice Archie Campbell in his assessment of the Ontario SARS outbreak[46]. Particularly, this province-wide system would address issues surrounding a lack of public health surge capacity. As an early warning system, Telehealth has the potential to identify aberrations long before traditional reporting systems. In the case of the Walkerton, Ontario outbreak[47], if Telehealth had been up and operational as an early warning system, we can speculate that the number of calls for gastrointestinal complaints would have gone up, which would have created an alert for public health to investigate these calls before an increase in cases would have been identified through routine reporting systems (especially RDIS). By acting early, the levels of morbidity and mortality witnessed in Walkerton could have been curtailed by a more timely involvement of Public Health. This would have meant that control measures such as "boil water" orders) could have implemented more quickly, which would have decreased the number of cases and would have allowed public health to work more effectively and within its available resources.

This system would also improve communication and coordination between the different levels of government (especially regional and provincial) by centralizing and streamlining the information flow, and potentially identifying important health events before they are identified by routine surveillance activities. This would also allow for the implementation of emergency plans (e.g. antiviral distribution in the event of pandemic influenza) before the needs placed on public health and the acute care centers exceeds available resources, thereby enhancing the effectiveness of medical practice. This has both cost- and life-saving implications, as the Ontario SARS experience has shown us.

This surveillance system is also unique and innovative in that it does not rely on reporting of cases by physicians. In other words, it allows for the surveillance of underserviced areas such as northern Ontario, as the system relies on individuals calling a toll-free number rather than trying to get access to a physician who then has a reporting responsibility. In effect, for the purpose of surveillance, it cuts out the need for a physician as a "reporting" intermediary. This will potentially allow for a more complete picture of underserviced areas, as well as, a much timelier one as it is available 24 hours a day, seven days a week, 365 days a year. The continuous availability of this service provides a front line that each and every Ontarian can access prior to or instead of seeking help from a family physician or an emergency department. When a call is placed to Telehealth, all callers are given advice based on the type and severity of symptoms they are reporting. Advice can range from staying at home to going to an emergency department immediately. Because of its province-wide coverage, this means that data for areas with limited access to emergency departments and family physicians will be collected, thereby providing new information on an underserviced subset of the population.

Furthermore, areas such as aboriginal communities have health clinics, often staffed with nurse practitioners. However, these clinics are not integrated with routine surveillance systems. Use of Telehealth for surveillance would also allow for isolated communities with few health resources to be included into a more formal surveillance system.

Finally, this project is important and of value because it has the potential to show the different levels of government that province-wide surveillance that relies on the integration of available data sources into the surveillance information flow is feasible, cost-effective, and has the potential to positively impact morbidity and mortality through better planning and the subsequent enhanced effectiveness of public health practice.

## List of abbreviations

CAS Clinical Assessment System

ED Emergency Department

HMO Health Management Organization

ICD International Classification of Diseases

NHS National Health Service (United Kingdom)

OTC Over the counter

QUESST Queen's University Emergency Syndromic Surveillance Team

RDIS Reportable Disease Information System (Ontario, Canada)

SARS Severe Acute Respiratory Syndrome

## Competing interests

The author(s) declare that they have no competing interests.

## Authors' contributions

ER participated in the design of the study and drafted the manuscript.

KMM conceived of the study, and participated in its design and helped to draft the manuscript.

VR and DM helped to draft the manuscript.

All authors read and approved the manuscript.

## Pre-publication history

The pre-publication history for this paper can be accessed here:



## Figures and Tables

**Table 1:** Syndrome Categories

**Syndrome**	**Definition**
Gastrointestinal	pain or cramps anywhere in the abdomen nausea, vomiting, diarrhea, and abdominal distension and swelling.
Constitutional	non-localized, systemic problems including fever, chills, body aches, flu symptoms (viral syndrome), weakness, fatigue, anorexia, malaise, lethargy, sweating (diaphoresis), light headedness, faintness and fussiness.
Respiratory	problems of the nose (coryza) and throat (pharyngitis), as well as the lungs. Examples of respiratory include congestion, sore throat, tonsillitis, sinusitis, cold symptoms, bronchitis, cough, shortness of breath, asthma, chronic obstructive pulmonary disease (COPD), and pneumonia. The presence of both cold and flu symptoms is counted in this category, not Constitutional.
Rash	any rash, such as macular, papular, vesicular, petechial, purpuric, or hives. Ulcerations are not counted as Rash unless consistent with cutaneous anthrax (an ulcer with a black eschar).
Hemorrhagic	bleeding from any site, e.g., vomiting blood (hematemesis), nose bleed (epistaxis), hematuria, gastrointestinal bleeding (site unspecified), rectal bleeding, and vaginal bleeding. Bleeding from a site for which there is a syndrome is counted as Hemorrhagic and as the relevant syndrome (e.g. hematochesia is Gastrointestinal and Hemorrhagic; hemoptysis is Respiratory and Hemorrhagic).
Botulinic	ocular abnormalities (diplopia, blurred vision, photophobia), difficulty speaking (dysphonia, dysarthria, slurred speech), and difficulty swallowing (dysphagia).
Neurological	non-psychiatric complaints that relate to brain function. Included are headache, head pain, migraine, facial pain or numbness, seizure, tremor, convulsion, loss of consciousness, syncope, fainting, ataxia, confusion, disorientation, altered mental status, vertigo, concussion, meningitis, stiff neck, tingling and numbness. (Dizziness is both Constitutional and Neurological).
Other	anything which does not fall into any of the above categories, particularly injuries.
